# Circularly polarized light detection with hot electrons in chiral plasmonic metamaterials

**DOI:** 10.1038/ncomms9379

**Published:** 2015-09-22

**Authors:** Wei Li, Zachary J. Coppens, Lucas V. Besteiro, Wenyi Wang, Alexander O. Govorov, Jason Valentine

**Affiliations:** 1 Department of Mechanical Engineering, Vanderbilt University, Nashville, Tennessee 37212, USA; 2 Department of Physics and Astronomy, Ohio University, Athens, Ohio 45701, USA; 3 Department of Electrical Engineering, Vanderbilt University, Nashville, Tennessee 37212, USA

## Abstract

Circularly polarized light is utilized in various optical techniques and devices. However, using conventional optical systems to generate, analyse and detect circularly polarized light involves multiple optical elements, making it challenging to realize miniature and integrated devices. While a number of ultracompact optical elements for manipulating circularly polarized light have recently been demonstrated, the development of an efficient and highly selective circularly polarized light photodetector remains challenging. Here we report on an ultracompact circularly polarized light detector that combines large engineered chirality, realized using chiral plasmonic metamaterials, with hot electron injection. We demonstrate the detector's ability to distinguish between left and right hand circularly polarized light without the use of additional optical elements. Implementation of this photodetector could lead to enhanced security in fibre and free-space communication, as well as emission, imaging and sensing applications for circularly polarized light using a highly integrated photonic platform.

## Abstract (web summary)

Analysis and detection of circularly polarized light involves the use of multiple optical elements. Here, the authors demonstrate an ultracompact circularly polarized light detector using chiral plasmonic metamaterials with hot electron injection, realizing its implementation on an integrated photonic platform.

## Graphical abstract

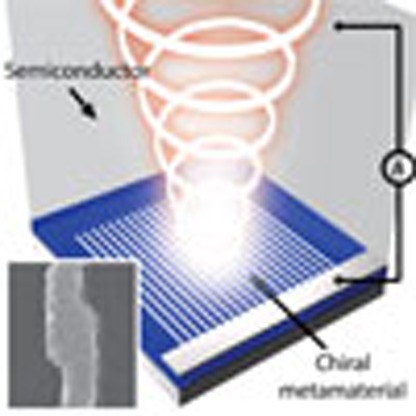

Circularly polarized light (CPL) is utilized in various optical techniques and devices, ranging from quantum computation^1^,^2^,^3^ and spin optical communication^4^ to circular dichroism (CD) spectroscopy^5^ and magnetic recording^6^. In CPL, the electric field vector travels along a helical trajectory, either clockwise or counterclockwise^7^, and it can be generated from two linearly polarized light (LPL) waves with perpendicular electric field vectors that are oscillating with a 90° phase shift. Distinguishing between the two polarizations of CPL is inherently difficult with conventional photodetectors due to the fact that conventional semiconductors lack intrinsic chirality. However, in nature, some species of mantis shrimp can detect CPL and use it as a private channel of communication that is unavailable to both predators and potential competitors^8^. The origin of this ability comes from specialized cells in the retina that serve as quarter-wave plates that sit atop LPL sensitive photoreceptors and convert CPL into LPL^8^. This is done in the same manner as CPL is detected using conventional optics, namely, through the combination of a non-chiral photodetector with a quarter waveplate and a linear polarizer. While ultracompact optical elements and devices including CPL sources^9^,^10^, quarter waveplates^11^, polarizers^12^ and beam splitters^13^,^14^ have been successfully demonstrated, the use of multiple optical elements to distinguish CPL makes it challenging to realize miniature and integrated CPL detectors.

One alternative technique is the use of chiral media as the active material in a photodetector. A chiral medium, one in which the unit cell cannot be superimposed on its mirror image, responds differently for left-handed circularly polarized (LCP) light and right-handed circularly polarized (RCP) light. Chiral media exists in nature, for example, the iridescent metallic green beetle, *Chrysina gloriosa*, selectively reflects LCP light but absorbs RCP due to structural chirality^15^, while sugar molecules, acids and proteins exhibit intrinsic chirality. Recently, a chiral organic semiconductor transistor has been demonstrated for direct detection of CPL using the intrinsic chiral response of helicene at a wavelength of 365 nm (ref. 16). Although this technique shows promise, the organic semiconductor is unstable in ambient conditions. It also has a limited response time and the operational wavelength is limited to the ultraviolet regime.

Over the past decade, advances in plasmonics have provided a mechanism for strong light–matter interaction leading to strong field enhancement and resonant scattering and absorption. Furthermore, surface plasmon resonances offer new insights into creating artificial media, or metamaterials, with interesting optical properties such as strong chirality^17^,^18^ that is several orders of magnitude larger than that of chiral molecules. A number of chiral metamaterials based on plasmonic building blocks including spiral^19^, fish-scale metamaterials^20^, helix^12^,^21^,^22^, oligomers^23^ and twisted metamaterials^24^,^25^ have been demonstrated to date using both bottom-up^21^,^26^ and top-down approaches^12^,^23^,^24^,^25^. In addition, Archimedes spiral designs have been utilized to enable chiral-selective field enhancements in semiconductors, leading to selective photocurrent for LCP and RCP light, with an experimentally measured ratio of 1.13 (ref. 19). The use of plasmonic elements also opens the door to photon energy harvesting through hot carrier generation and injection^27^,^28^, leading to a new scheme for photodetection^29^,^30^,^31^,^32^,^33^,^34^,^35^ and photocatalysis^36^,^37^. Hot carriers generated from the non-radiative decay of surface plasmons can be captured with electron acceptors such as metal–semiconductor Schottky interfaces^29^,^30^,^31^,^32^,^35^,^38^ or metal–insulator–metal junctions^33^,^34^, resulting in photocurrent. This scheme enables direct conversion of light into an electrical signal-based solely on the photoexcited carriers generated from the absorption in plasmonic nanostructures. The tunability of the plasmonic structures enables a straightforward tailoring of the response of a photodetector including the working wavelength, bandwidth and polarization dependence^29^,^32^,^34^,^35^.

In the present work, we propose and experimentally demonstrate an ultracompact CPL detector in which the ability to distinguish LCP and RCP comes from the engineered chirality in plasmonic nanostructures. Photodetection is based on hot electron injection into silicon with operation in the telecommunications band. Implementation of this CPL photodetector could lead to enhanced security in fibre and free-space communication as well as emission, imaging and sensing applications for CPL.

## Results

### Chiral metamaterial and device design

**Figure 1:**
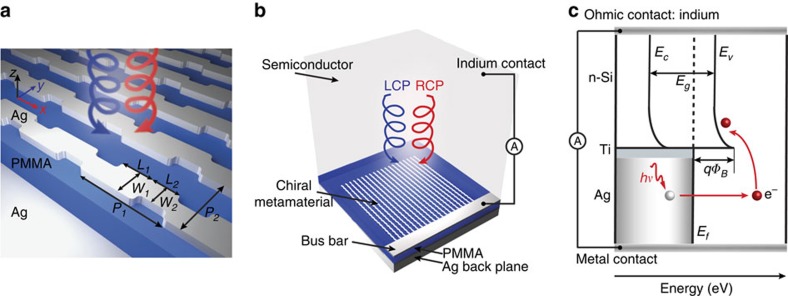
Schematic of the chiral metamaterial and the CPL detector. (**a**) Schematic of the chiral metamaterial consisting of the chiral plasmonic meta-molecule array, dielectric spacer and metal backplane. The dimensions of the meta-molecules are *L*_1_=125 nm, *L*_2_=105 nm, *W*_1_=115 nm, *W*_2_=85 nm, *P*_1_=335 nm and *P*_2_=235 nm. The thickness of the meta-molecules, dielectric spacer and the metal backplane are 40, 160 and 100 nm, respectively. (**b**) Schematic of the CPL detector consisting of a chiral metamaterial integrated with a semiconductor that serves as a hot electron acceptor. The Ohmic contact on Si is formed by soldering indium. The circuit is formed by wire bonding to the silver bus bar and indium. (**c**) Energy band diagram of the CPL detector. A Schottky barrier is formed between Si and the Ti interfacial layer. The hot electrons that are photo-generated in the Ag metamaterial are then injected over this barrier into the Si.

Our proposed chiral metamaterial (Fig. 1a) is a periodic array of chiral meta-molecules, with a unit cell consisting of a ‘Z'-shaped silver (Ag) antenna on top of a poly(methyl methacrylate) (PMMA) spacer and an optically thick Ag backplane. The unit cell shape of this chiral metamaterial allows for an inherent electrical connection among all the elements, forming nanowires, while a silver bus bar is used to electrically connect the nanowires (Fig. 1b). The whole device is realized by placing an n-type silicon wafer in contact with the antenna layer, forming a Schottky barrier (Fig. 1c). Light is incident on the frontside of the Si wafer, transmitting to the backside where the chiral metamaterial absorbs photons of a particular handedness, generating electrons within the metal at higher energy states. The energetic electrons (or hot electrons) with energy higher than the Schottky barrier can emit over the Schottky interface, leading to a detectable current (Fig. 1c).

**Figure 2:**
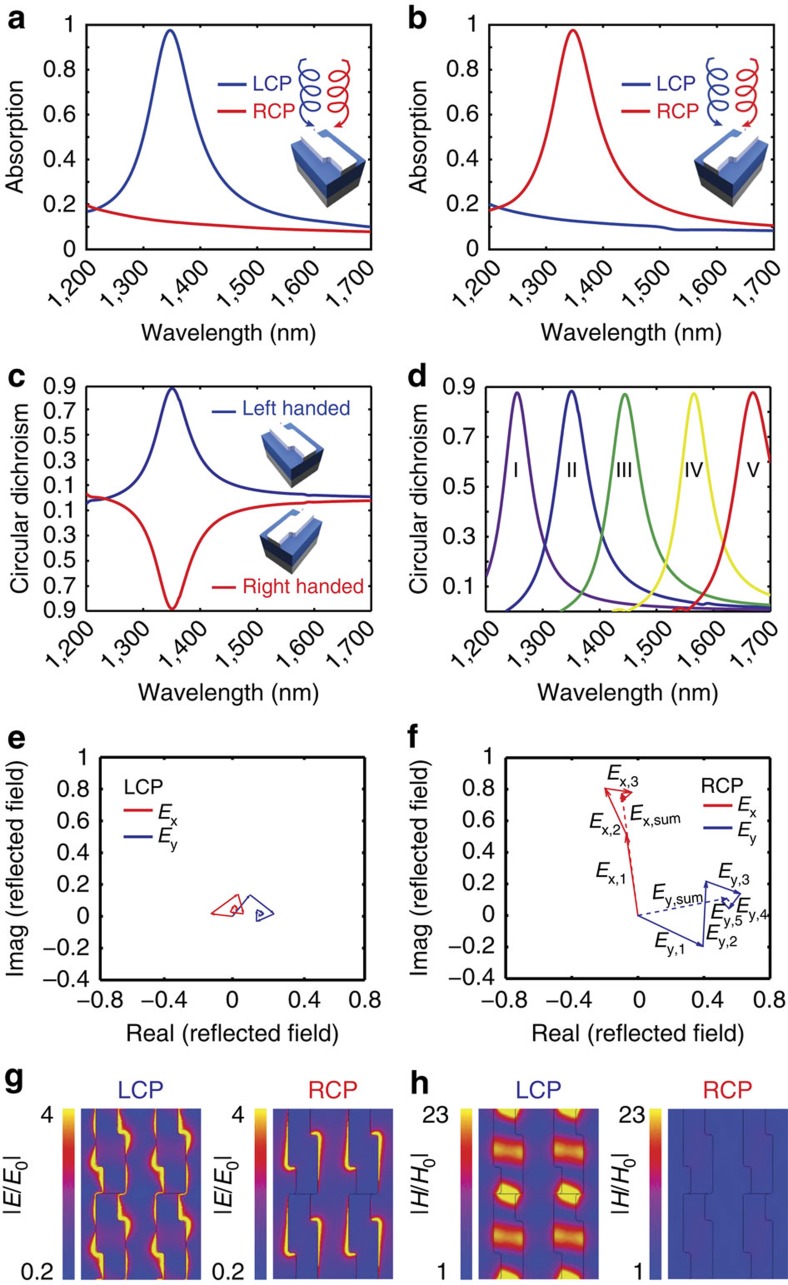
Simulated optical response of chiral metamaterial. (**a**,**b**) Simulated optical absorption spectra under LCP (blue) and RCP (red) illumination for LH (**a**) and RH (**b**) metamaterials. (**c**) Circular dichroism spectra for both the LH (blue) and RH (red) metamaterial. (**d**) CD as a function of resonator size. Dimensions of the structures (I–V) are follows: *L*_1_=115, 125, 130, 150 and 160 nm; *L*_2_=95, 105, 120, 130 and 140 nm; *W*_1_=110, 115, 120, 120 and 140 nm; *W*_2_=85, 85, 90, 90 and 100 nm; *P*_1_=305, 335, 370, 410 and 440 nm; *P*_2_=230, 235, 240, 240 and 260 nm, respectively. The other dimensions are the same as Fig. 1a. (**e**,**f**) For the LH metamaterial, the reflected LPL components, *E*_*x*_ (red) and *E*_*y*_ (blue), on multiple reflections for LCP (**e**) and RCP (**f**) light at wavelength of 1,350 nm. (**g**,**h**) Simulated electric (**g**) and magnetic (**h**) fields for LCP and RCP illumination at wavelength of 1,350 nm.

To investigate the optical response of the device on CPL illumination, full-wave electromagnetic simulations were first performed. LCP and RCP light are incident onto the frontside of the silicon wafer, transmitting to the backside where the chiral metamaterial selectively absorbs light with a particular handedness while reflecting the other. For example, a left-handed (LH) chiral metamaterial acts as a perfect LCP light absorber at the resonant wavelength while reflecting nearly 90% of the RCP light (Fig. 2a) and vice versa for the right-handed (RH) chiral metamaterial (Fig. 2b), leading to a CD (CD=*A*_LCP_−*A*_RCP_) of almost 0.9 (Fig. 2c). Replacing the antenna layer with an unstructured Ag film results in 95% reflection, indicating we are nearly at the Drude damping limit. The chiral response can also be tuned across the entire telecommunication regime as shown in Fig. 2d. The minimum and maximum working wavelengths are only limited by the semiconductor bandgap energy and the Schottky barrier height, respectively. The limit imposed by silicon's bandgap is due to the fact that we are illuminating the device through the silicon wafer and must avoid direct absorption by the semiconductor.

To understand the origin of the strong CD, the CPL electric field vector can be decomposed as two orthogonal LPL electric field vectors, *E*_*x*_ and *E*_*y*_, that are oscillating with a 90° phase shift:


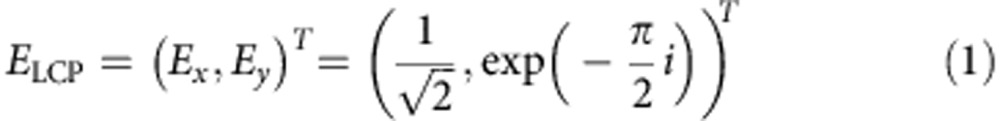



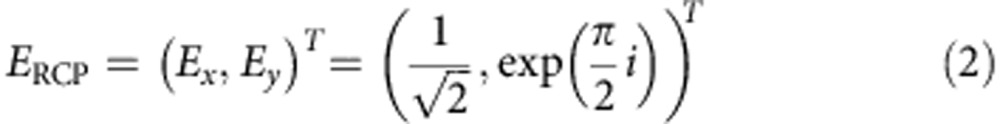


These fields are input into a multiple reflection model in which we track the reflected electric field evolution as a function of the number of reflections (Fig. 2e,f) between the LH chiral metamaterial and the metal backplane. As seen in Fig. 2e,f, on multiple reflections, both the reflected *E*_*x*_ and *E*_*y*_ fields for LCP are diminished (Fig. 2e), while the RCP fields grow (Fig. 2f). This highly asymmetric effect (Fig. 2g,h) comes from the destructive (LCP) and constructive (RCP) interference of the illumination beams and relies on the fact that the planar metamaterial is lossy, anisotropic, and results in linear polarization conversion. More details regarding these requirements, the response of this specific metamaterial, and general design guidelines for planar chiral metamaterials can be found in the Supplementary Information (Supplementary Figs 1–6; Supplementary Notes 1 and 2). It is also important to note that while the handedness of CPL is reversed at the metal backplane the reflected light will see the handedness of the planar chiral metamaterial reversed as well.

### Photodetector performance

**Figure 3:**
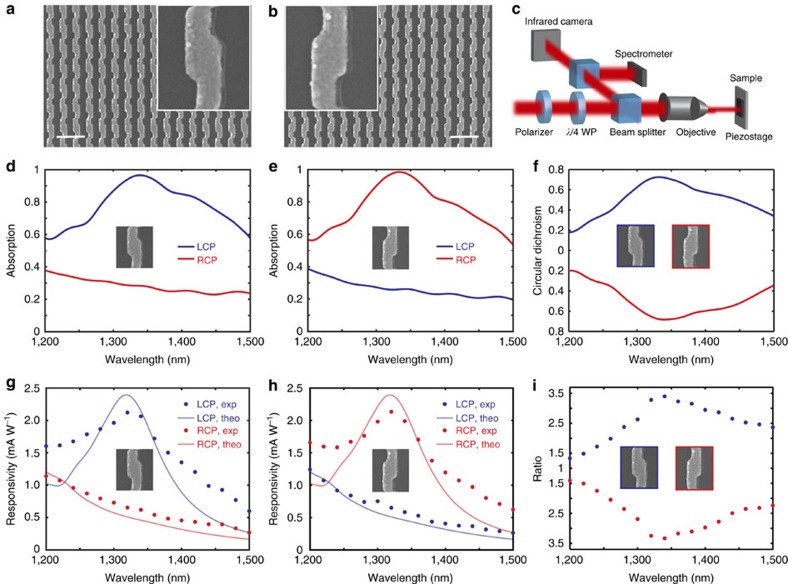
Experimentally measured optical absorption and photoresponsivity spectra. (**a**,**b**) Scanning electron microscope images of the LH (**a**) and RH (**b**) metamaterial before spin coating the PMMA spacer layer. The inset shows a unit cell of the chiral metamaterial. Scale bar, 500 nm. (**c**) Schematic of experimental set-up. (**d**,**e**) Experimentally measured optical absorption spectra under LCP (blue) and RCP (red) illumination for LH (**d**) and RH (**e**) metamaterials. (**f**) Experimentally measured circular dichroism spectra for both LH (blue) and RH (red) metamaterials. (**g**,**h**) Experimentally measured (dots) and theoretically calculated (solid curve) photoresponsivity spectra under LCP (blue) and RCP (red) illumination for LH (**g**) and RH (**h**) metamaterials. (**i**) Photocurrent polarization discrimination ratio spectra of LH and RH metamaterials. The metamaterials measured have overall areas of 70 × 70 μm^2^.

The high CD directly leads to enhanced discrimination between LCP and RCP in the CPL photodetectors. To demonstrate this, we fabricated both LH and RH chiral metamaterial arrays (Fig. 3a,b) on the bottom side of a double-side polished n-type silicon wafer (see the Methods section). The double-side polished wafer is chosen to allow light illumination from the frontside of the wafer without inducing scattering. A Schottky interface is formed between the chiral plasmonic metamaterial and the semiconductor substrate, allowing us to capture the generated hot electrons. The plasmonic metamaterial was fabricated by first depositing a 1-nm-thick titanium layer (forming the Schottky barrier) followed by a 45-nm-thick aluminum-doped silver film^39^. Aluminum-doped silver was used due to the fact that it helps to reduce the silver film roughness and grain size while also facilitating the formation of an Al_2_O_3_ passivation layer that protects the silver from corrosion^39^.

The experimentally measured optical absorption spectrum of both the LH and RH metamaterial reveals the same absorption bands demonstrated in the simulations. For the LH metamaterial, there is a resonance leading to near unity absorption of LCP light at 1,340 nm, whereas RCP light is largely reflected (Fig. 3d). The RH metamaterial shows a nearly opposite optical response (Fig. 3e). One will notice that the off-resonant absorption is increased compared with the simulation. This is due to the increased material loss, induced by the aluminum doping of silver and the interfacial 1-nm Ti layer. Even with the slightly increased off-state absorption, the metamaterial still possesses a CD of 0.72 at 1,340 nm (Fig. 3f), among the highest reported for chiral metamaterials.

Photoresponse spectra of the devices were obtained by illuminating the metamaterials with a circularly polarized laser and measuring photocurrent as a function of the laser handedness and wavelength. The photoresponsivity spectrum (Fig. 3g,h) matches well with the measured absorption spectrum. The peak photoresponsivity of the resonant state reaches 2.2 mA W^−1^, corresponding to a quantum efficiency of ∼0.2% which is comparable to other Schottky diode-based LPL photodetectors working in this wavelength regime^32^,^35^. This efficiency is two times that of chiral organic semiconductor transistors^16^ and could be further improved by embedding the resonator layer in the silicon^31^. Furthermore, the large CD translates to a correspondingly large distinction in the photocurrent for LCP and RCP light with a polarization discrimination ratio of 3.4 (Fig. 3i) and a difference in photoresponsivity of 1.5 mA W^−1^, demonstrating the ability to detect and distinguish between left and right hand CPL in an ultracompact detector geometry. It should be noted that the photoresponsivity of this device under non-CPL excitation is about 1.4 mA W^−1^, which is the average value of the resonant and off-resonant polarization state responsivity due to the fact that LPL can be decomposed into two CPL beams with opposite handedness. It is also important to note that although our device responsivity is still low compared with non-chiral InGaAs or Ge detectors (∼900 mA W^−1^) in this wavelength range, the later is not able to distinguish CPL. A similar functionality could be achieved by integrating the chiral metamaterial with such semiconductors, however, this does not necessarily produce high polarization selectivity^19^ (Supplementary Fig. 7; Supplementary Note 3) even though a higher quantum efficiency could be realized.

The theoretical photoresponsivity spectra were calculated (solid curve in Fig. 3g,h) based on the creation of energetic electrons due to the collisions with the interface potential wall between the metal and the silicon substrate^40^,^41^. Local injection current maps were calculated (Supplementary Fig. 8) and the total injected current is obtained by integrating over the Schottky interface (see the Methods section). The general trend of the experimental measurements and the theoretically calculated photoresponsivity spectra agrees well, although a resonance broadening is observed in the experimental results. This follows well with the inhomogeneous broadening of the experimentally measured optical absorption spectra compared with simulations (Supplementary Figs 9 and 10), and is most likely due to fabrication imperfections in the metamaterial.

**Figure 4:**
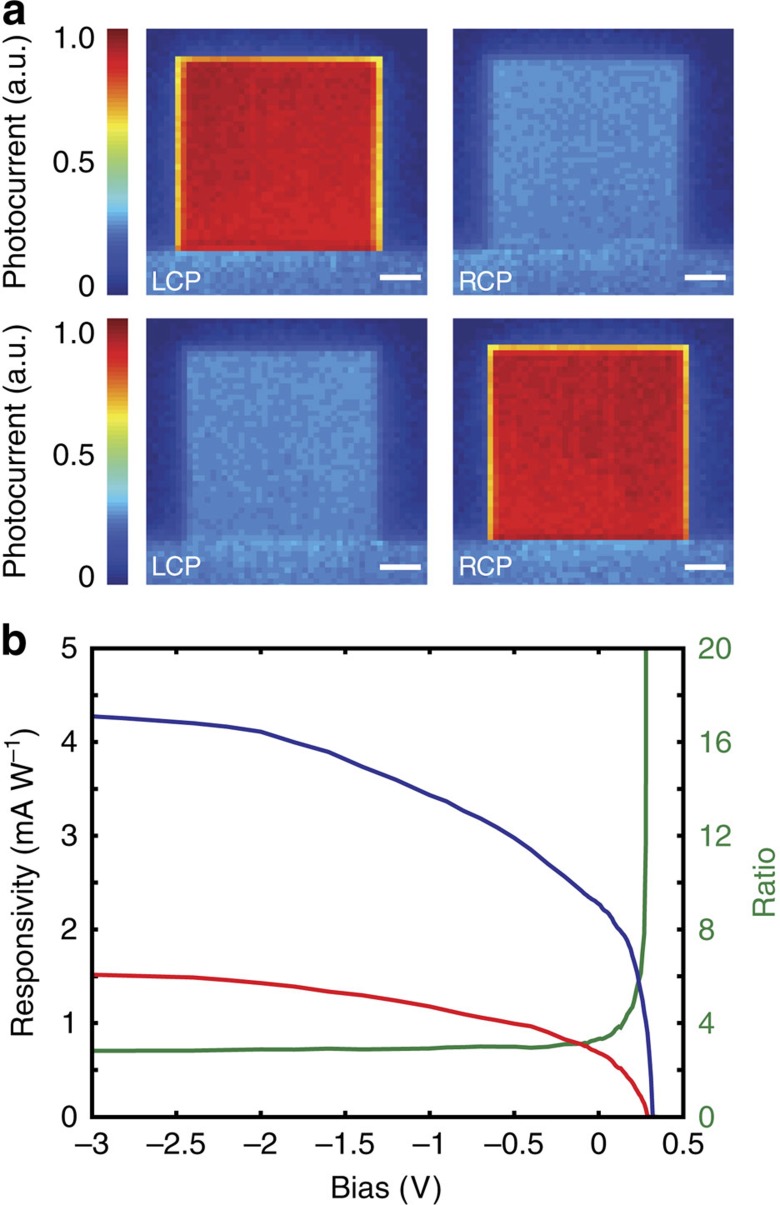
Spatial scanning and bias-dependent photocurrent. (**a**) Scanning photocurrent map of LH (top) and RH (bottom) metamaterials under LCP and RCP illumination. Scale bar, 15 μm. (**b**) Bias dependency of photocurrent of a LH metamaterial for LCP (blue) and RCP (red) light under a laser power of 1.5 mW. The green curve shows the polarization discrimination ratio versus bias.

Photocurrent maps were also measured by scanning a focused circularly polarized laser across the array and monitoring photocurrent as a function of spatial position. As can be seen in Fig. 4a, clear distinctions in photocurrent for LCP and RCP light are observed for both LH and RH metamaterials. The photocurrent of the chiral metamaterials under the off-resonant polarization is similar to the un-patterned silver bus bar, which confirms the non-resonant behaviour of the structure. The off-array photocurrent is within the noise level of the measurements, which confirms that direct absorption in the silicon is negligible. Furthermore, photocurrent measurements under different laser powers (Supplementary Fig. 11) show good linearity, excluding any non-linear interactions. It also indicates the detector's ability to work over a wide range of incident power, overcoming saturation issues found in chiral organic semiconductors^16^.

As an added benefit, Schottky diode-based photodetectors can provide photocurrent tuning through the application of a source–drain bias to the device^30^,^31^,^33^,^34^ offering flexibility in controlling the polarization discrimination ratio and photoresponsivity. As shown in Fig. 4b, when a negative bias was applied, both the LCP and RCP photocurrent were increased, leading to an increased difference in photoresponsivity. When a positive bias was applied, despite the decrease in absolute photocurrent, the polarization discrimination ratio or the selectivity can be markedly increased. These measurements indicate how one can generally trade photoresponsivity for a larger polarization discrimination ratio, or vice versa, to meet different application requirements.

**Figure 5:**
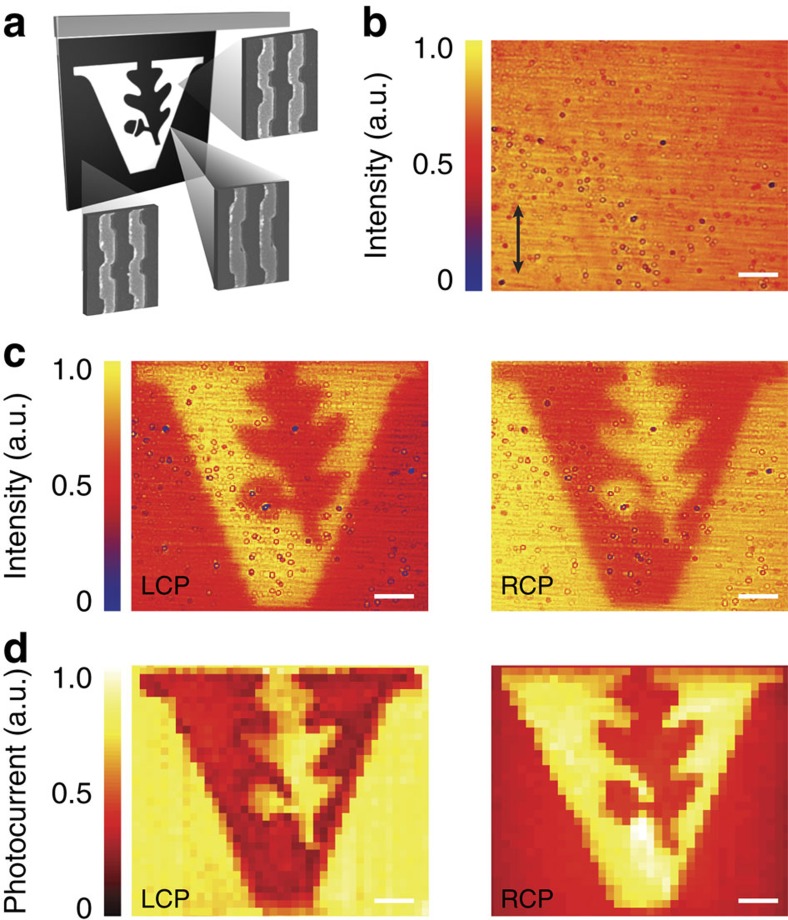
CPL detector with RH and LH elements patterned into the Vanderbilt University logo. (**a**) Schematic of the pattern with the LH metamaterial filling the black region and the RH metamaterial filling the white region. (**b**) Camera image of the metamaterial under linearly polarized light with polarization along the vertical direction. (**c**) Camera images of the metamaterial under LCP (left) and RCP (right) illumination. (**d**) Scanning photocurrent maps of the metamaterial under LCP (left) and RCP (right) illumination. Scale bar, 10 μm.

Since the chirality of our metamaterial originates from the structure of the antenna layer, instead of an intrinsic material chirality, multiple meta-molecules can be incorporated into a single device to achieve multiple functionalities including CPL, LPL and wavelength selectivity, with only one patterning step. As a proof of concept, we placed LH and RH chiral meta-molecules into a single array to form a spatially non-uniform, pixelated photodetector. The fabricated array is 90 × 90 μm, containing roughly 100,621 unit cells. The array was implemented with both LH and RH enantiomers that were patterned to create the Vanderbilt University logo (Fig. 5a), where the LH and RH enantiomers fill the black and white regions of the logo, respectively. The shape of our chiral meta-molecules also allows for an electrically connected transition between the opposite-handed enantiomers. As a result, an image of the ‘V' logo, which does not appear under linearly polarized (Fig. 5b) or un-polarized light, shows a very clear contrast in reflection under LCP and RCP light (Fig. 5c). The performance of this integrated detector was evaluated using scanning photocurrent measurements with focused circularly polarized laser illumination. Good contrast is observed in the photocurrent scanning maps for LCP and RCP (Fig. 5d), indicating the ability to integrate multiple chiral plasmonic meta-molecules into a single-ultracompact device.

## Discussion

In summary, we have demonstrated a CPL detector based on the engineered CD in plasmonic nanostructures and the hot electron transfer process. This solid-state, hot electron-based chiral metamaterial CPL detector holds the promise of integration, robust and tunable operation^29^,^32^,^35^, and a large polarization discrimination. Combining our CPL detector with current existing linearly polarized light hot electron photodetectors^29^,^32^,^34^,^35^ could lead to an integrated hot electron polarimeter with the ability to determine the Stokes parameters or the states of polarization of an arbitrarily polarized beam^19^. These CPL detectors could be used in applications ranging from encoded fibre optic and free-space communications to polarimetric imaging, emission and sensing applications using CPL.

## Methods

### Simulations

Full-wave electromagnetic simulations were performed using commercially available software (CST Microwave Studio 2013 and Lumerical). In all simulations, periodic boundary conditions were used along the *x* and *y* axes and perfectly matched layers were used along the propagation directions. The simulations were performed using an n-type Si half-space above the metamaterial layer followed by the PMMA spacer layer and the silver backplane. The top simulation domain is terminated at silicon such that the reflection from the frontside of the silicon is not taken into account. In the proposed design (Fig. 1a), the optical parameters for Ag were taken from Johnson and Christy^42^ and the damping was increased by three times. In the experimental section, the optical parameters for Al-doped silver and n-type Si substrate were taken from ellipsometry measurements.

### Injection current calculation

In the theoretical treatment, photocurrent arises from the hot electrons that are generated near the metal–semiconductor interface by the plasmonic electric field that is normal to the interface^40^,^41^. The total injected current from the unit cell is given by


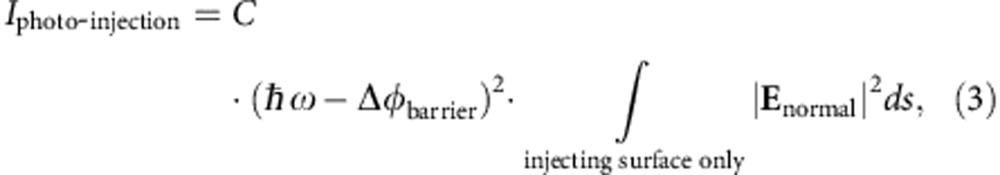


where the integral is taken over the metal–Si interface within the unit cell, *C* is a material constant depending on the Fermi energy and the injecting barrier height, and (*ħω*−Δ*φ*_Barrier_)^2^ is a factor reflecting the Fowler law (Supplementary Fig. 10; Supplementary Note 4). The barrier height was measured to be 0.54 eV (Supplementary Fig. 12). The constant *C* can be estimated from quantum theory and has a characteristic frequency dependence ∼1/*ω*^4^, which comes from the summation over amplitudes of quantum transitions.

### Sample fabrication

The device was fabricated on the bottom side of a double-side polished <100> n-type silicon (Si) wafer (500-μm thick, 1–10 Ωcm). After spin coating the substrate with 130-nm-thick PMMA, the antenna arrays were defined using electron beam lithography. The LH and RH arrays had an overall area of 70 × 70 μm^2^ and the VU Logo array was 90 × 90 μm^2^. After cold development in methyl isobutyl ketone (MIBK):isopropyl alcohol (IPA) 1:3, the sample was cleaned with an IPA rinse and dried under a N_2_ stream. The sample was immersed in 10:1 buffered oxide etchant for 10 s to completely remove the native oxide from the Si. It was then immediately transferred into an evaporation chamber followed by evaporation of 1 nm Ti and co-deposition of a 45-nm-thick Al-doped silver film. The interfacial Ti layer produces a Schottky barrier height of *φ*_B_=0.54 eV. The silver deposition rate was 2 Å s^−1^ and the aluminum deposition rate was 0.2 Å s^−1^, leading to an aluminum concentration of ∼9%. After the co-deposition a lift-off process was used to yield the structures shown in Fig. 3a,b. The obtained meta-molecule dimensions are *L*_1_=130 nm, *L*_2_=110 nm, *W*_1_=110 nm, *W*_2_=80 nm, *P*_1_=350 nm and *P*_2_=230 nm. Finally, a 160-nm-thick PMMA spacer layer was created by spin coating and followed by thermal evaporation of a 100-nm-thick silver backplane.

### Characterization

The optical absorption and CD spectrum were obtained using a broadband white-light source and a custom infrared microscope coupled with a grating spectrometer (Horiba, iHR320). CPL was generated with a polarizer combined with a quarter waveplate. The quality of the CPL was checked with an additional polarizer. To measure the reflection spectra, a silver mirror was used as the reference. The reflection from the frontside of the wafer, measured using an un-patterned single-side polished silicon wafer with same doping level, is subtracted from the reflection from the patterned sample. This matches the configuration in the simulations. The transmission is zero due to the optically thick silver backplane. An Ohmic contact on the n-type silicon substrate was achieved by indium soldering. The sample was wire bonded to a chip carrier to perform the photocurrent measurements. The photocurrent measurements were obtained using a custom scanning photocurrent microscope along with a tunable laser (Coherent Mira OPO), digital source meter (Keithley 2400), lock-in amplifier (SR830) and pre-amplifier (DL Instruments 1211). The laser was mechanically chopped (Thorlabs MC2000 & MC1F10) at 950 Hz with a lock-in integration time of 300 ms. Photocurrent maps were generated by raster scanning a focused circularly polarized laser (∼2 μm full-width at half-maximum) across the array and monitoring photocurrent as a function of spatial position. The scan step size is 2 μm in the *x* and *y* directions and for each measurement we dwell 1 s to stabilize the piezoelectric stage before recording data.

## Additional information

**How to cite this article:** Li, W. *et al.* Circularly polarized light detection with hot electrons in chiral plasmonic metamaterials. *Nat. Commun.* 6:8379 doi: 10.1038/ncomms9379 (2015).

## Supplementary Material

Supplementary Information Supplementary Figures 1-12, Supplementary Notes 1-4 and Supplementary References
