# Supplementary material for: Circularly polarized light detection with hot electrons in chiral plasmonic metamaterials
Source: Nat Commun. 2015 Sep 22;6:8379. doi: 10.1038/ncomms9379 (PMC4595755; doi:10.1038/ncomms9379)
Supplement: Supplementary Information — Supplementary Figures 1-12, Supplementary Notes 1-4 and Supplementary References [file ncomms9379-s1.pdf]

Supplementary Figures:

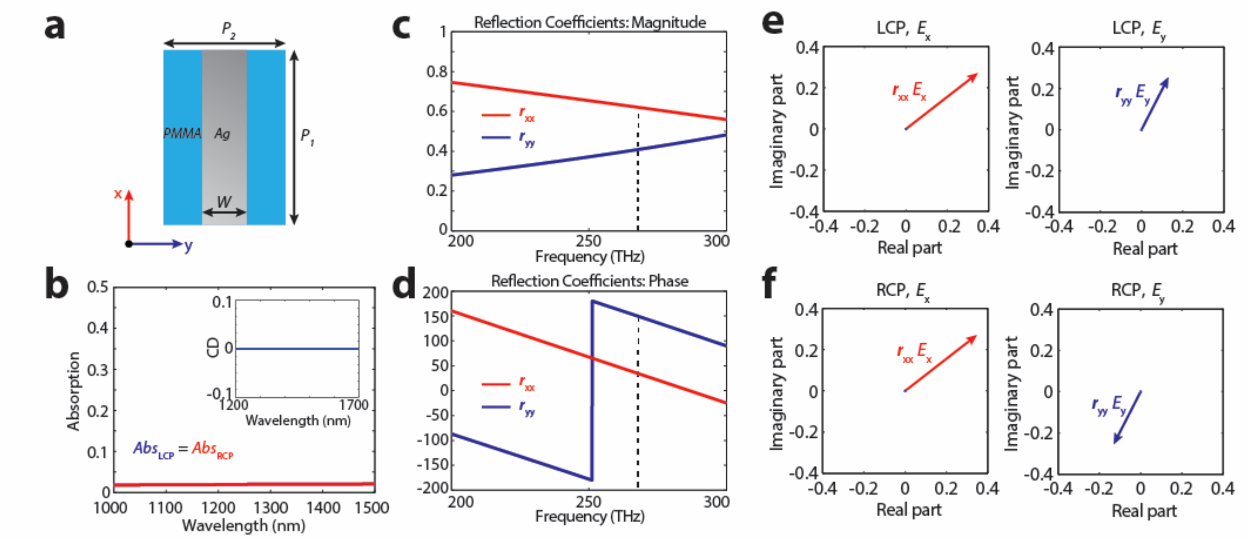

Supplementary Figure 1: Optical response of silver stripes without a metal ground plane. a, Schematic of a unit cell consisting of a silver stripe. Dimensions are P_1 = 630 nm, P_2 = 440 nm, W = 160 nm and the thickness of the silver stripes is 40 nm. Background material is PMMA (n = 1.47). **b,** Absorption spectra of LCP and RCP light. Inset shows the CD spectra. **c,d,** Amplitude (**c**) and phase (**d**) of reflection coefficients for E_x and E_y. **e,f,** Vector plots of the reflected fields $r_{xx}E_x$ and $r_{yy}E_y$ under LCP (**e**) and RCP (**f**) illumination at a frequency of f = 267 THz.

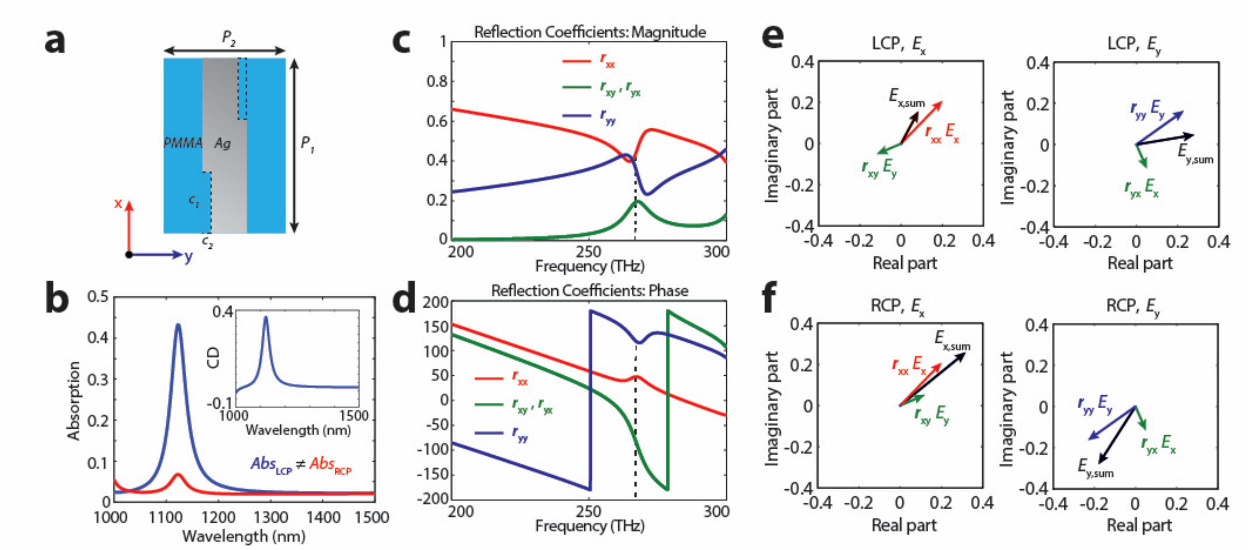

Supplementary Figure 2: Optical response of 'Z' shaped metamaterial without a metal ground plane. a, Schematic of of a 'Z' shaped antenna unit cell. The dimensions are $C_1 = 220$ nm and $C_2 = 30$ nm. The other dimensions are the same as Supplementary Fig. 1a. Background material is PMMA (n = 1.47). **b,** Absorption spectra of LCP and RCP light. Inset shows the CD spectra. **c,d,** Amplitude (**c**) and phase (**d**) of the reflection coefficients for E_x and E_y. **e,f,** Interference between the unconverted scattered field, $r_{xx}E_x$, (or $r_{yy}E_y$) and the converted scattered field, $r_{xy}E_y$ (or $r_{yx}E_x$), under LCP (**e**) and RCP (**f**) illumination at a frequency of $f = 267$ THz.

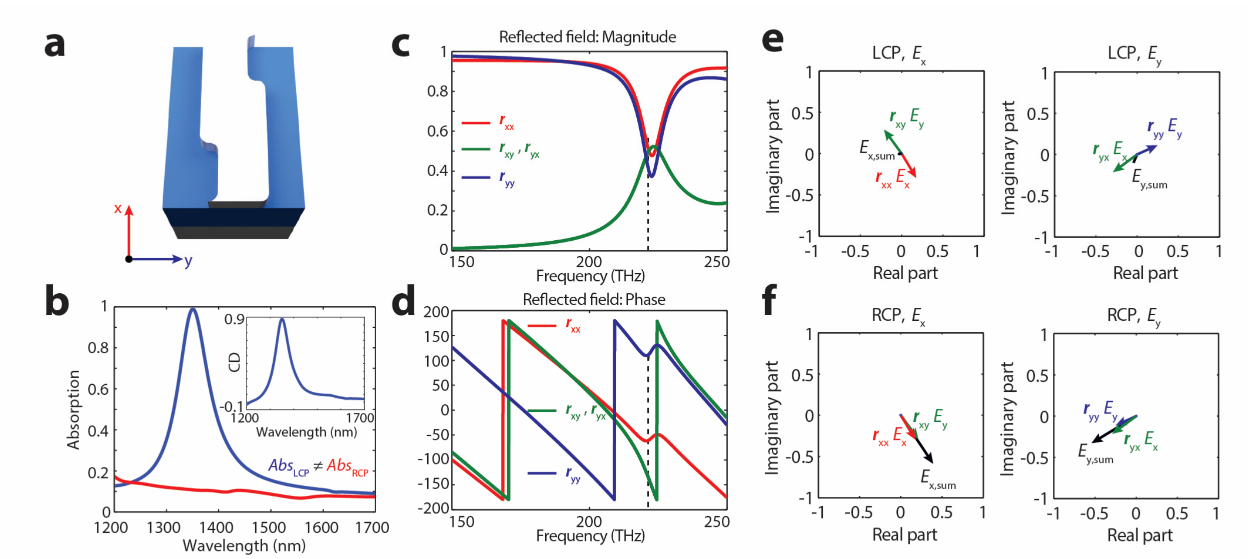

Supplementary Figure 3: Optical response of the 'Z' shaped chiral metamaterial with a metal ground plane. a, Schematic of a unit cell of the chiral metamaterial with metal ground plane, same as Fig.1a in the main text. **b,** Absorption spectra of LCP and RCP light. Inset shows the CD spectra. **c,d,** Amplitude (**c**) and phase (**d**) of the reflection coefficients for E_x and E_y. **e,f,** Interference between the unconverted scattered field, $\boldsymbol{r}_{xx}E_x$, (or $\boldsymbol{r}_{yy}E_y$) and the converted scattered field, $\boldsymbol{r}_{xy}E_y$ (or $\boldsymbol{r}_{yx}E_x$), under LCP (**e**) and RCP (**f**) illumination at a frequency of $f = 223$ THz.

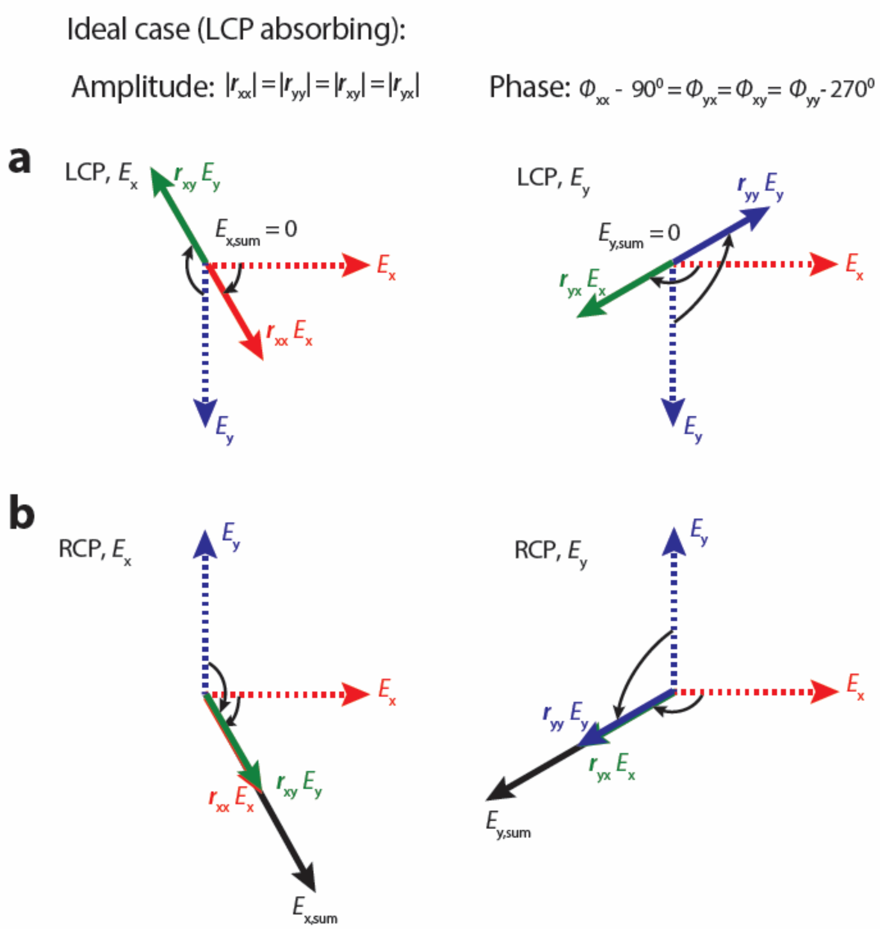

Supplementary Figure 4: Ideal conditions for maximizing circular dichroism in planar chiral metamaterials. Completely destructive (**a**) and constructive (**b**) interference between the unconverted scattered field, $\boldsymbol{r}_{xx}E_x$, (or $\boldsymbol{r}_{yy}E_y$) and the converted scattered field, $\boldsymbol{r}_{xy}E_y$ (or $\boldsymbol{r}_{yx}E_x$) under LCP (**a**) and RCP (**b**) illumination. Dashed lines represent the initial incident field vectors E_x and E_y with a 90° phase shift. Solid lines represent the reflected field vectors.

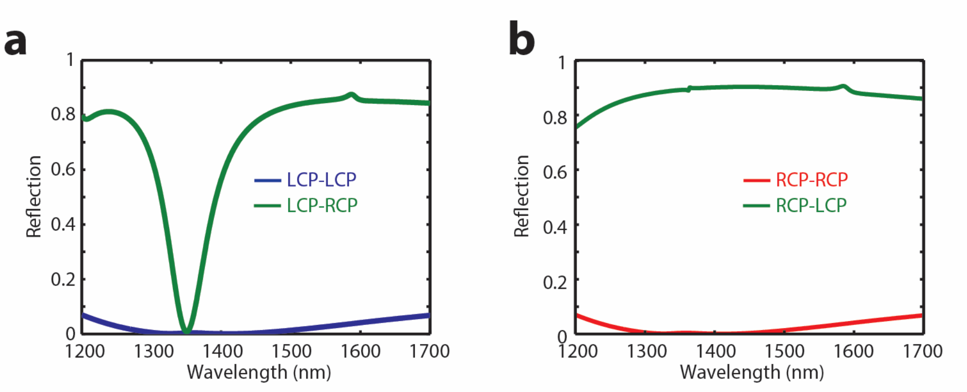

Supplementary Figure 5: Circular conversion dichroism of the chiral metamaterial. a, Under LCP illumination, the LCP reflection (blue) and LCP to RCP conversion (green) both go to minimum at the resonant wavelength, leading to the minimum total reflection field and maximum absorption. **b,** Under RCP illumination, the RCP reflection (green) is still minimum. However, there is a strong broadband polarization conversion from RCP to LCP (green). These simulations are performed on a LH metamaterial.

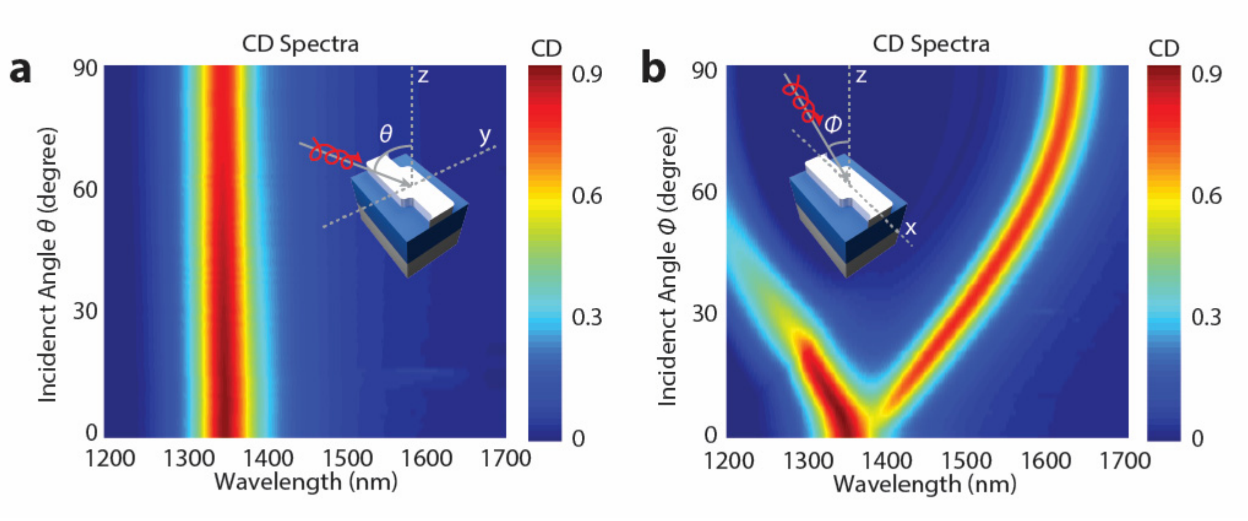

Supplementary Figure 6: Incident angle dependent circular dichroism for two different incidence directions. a, For incident light in the *yz* plane, the position of the CD peak is well maintained for all incident angles. **b,** For incident light in the *xz* plane, the CD spectra is maintained for a ~10° incident angle and further increasing the incident angle will shift the CD peak position. These simulations are performed on a LH metamaterial.

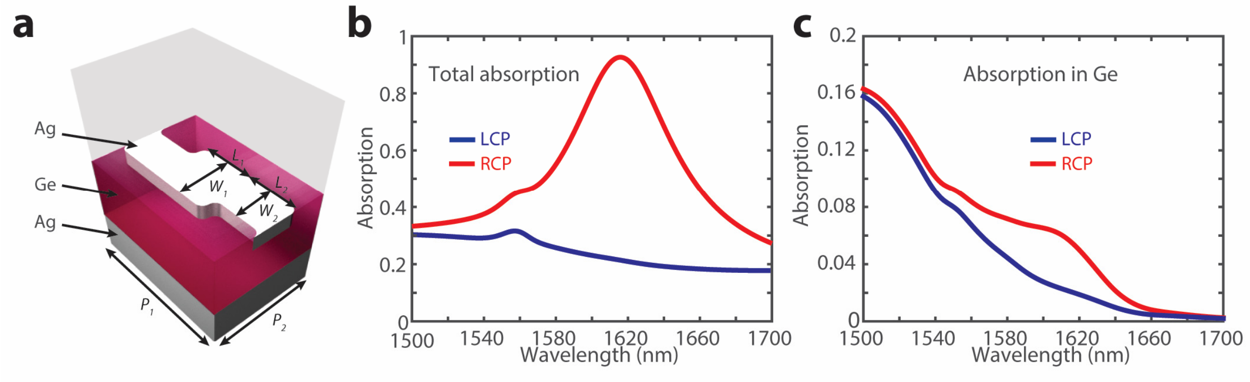

Supplementary Figure 7: Absorption spectra of the chiral metamaterial integrated with germanium. a, Schematic of the chiral metamaterial unit cell integrated with germanium. The dimensions are L_1 = 100 nm, L_2 = 110 nm, W_1 = 120 nm, W_2 = 80 nm, P_1 = 260 nm, P_2 = 340 nm. The thickness of the antenna, dielectric spacer and the metal back plane are 40 nm, 280 nm, and 100 nm respectively. **b,** Total absorption spectra of the chiral metamaterial for LCP (blue) and RCP (red) light. **c,** Absorption in germanium for LCP (blue) and RCP (red) light.

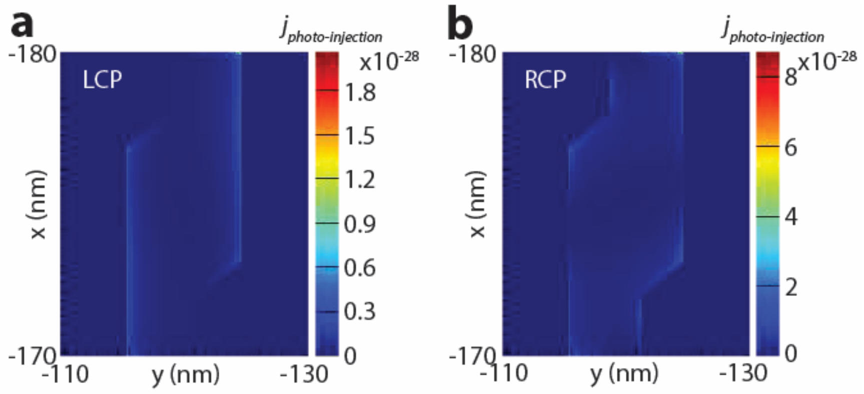

Supplementary Figure 8: Calculated maps of the local injection current. a, The local injection current map for a RH metamaterial under LCP illumination. **b,** The local injection current map for a RH metamaterial under RCP illumination. The theoretically modeled local injection current maps are given by the local normal electric field at the Schottky interface. The currents were calculated using, $j_{photo-injection}(r) \propto \left|E_{normal}\right|^2$. The total injection current is obtained by integrating over the Schottky interface. In this simulation, optical parameters for Al-doped silver were taken from ellipsometry measurements.

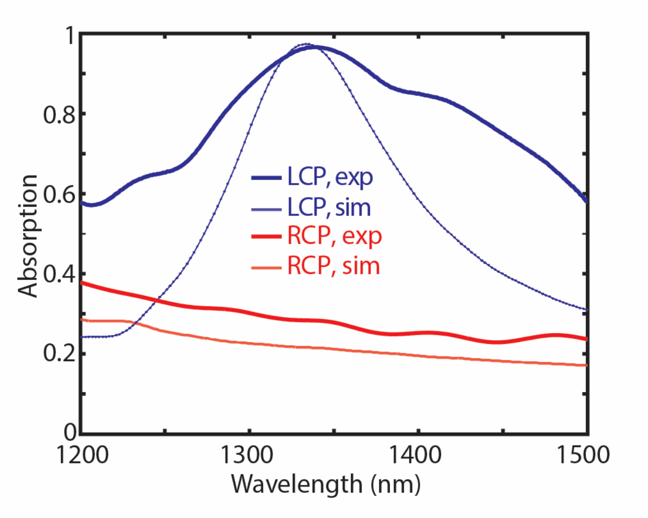

Supplementary Figure 9: Experimental measured and simulated optical absorption spectra of LH metamaterials. In the simulation, optical parameters for Al-doped silver were taken from ellipsometry measurements. One can see that a broadened resonant absorption is observed in the experimental results. This is attributed to the inhomogeneity in the chiral metamaterial arrays due to fabrication imperfections.

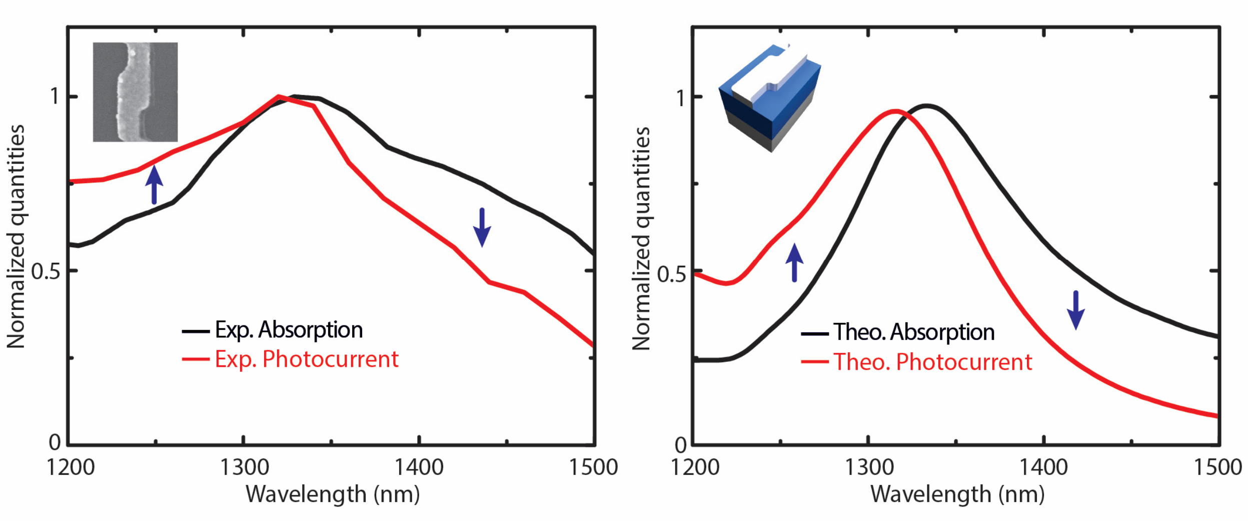

Supplementary Figure 10: Comparison of the absorption and photocurrent spectra. a, Normalized experimental measured absorption (black) and photocurrent (red) spectra for a RH metamaterial under RCP illumination. **b,** Normalized theoretical calculated absorption (black) and photocurrent (red) spectra for a RH metamaterial under RCP illumination.

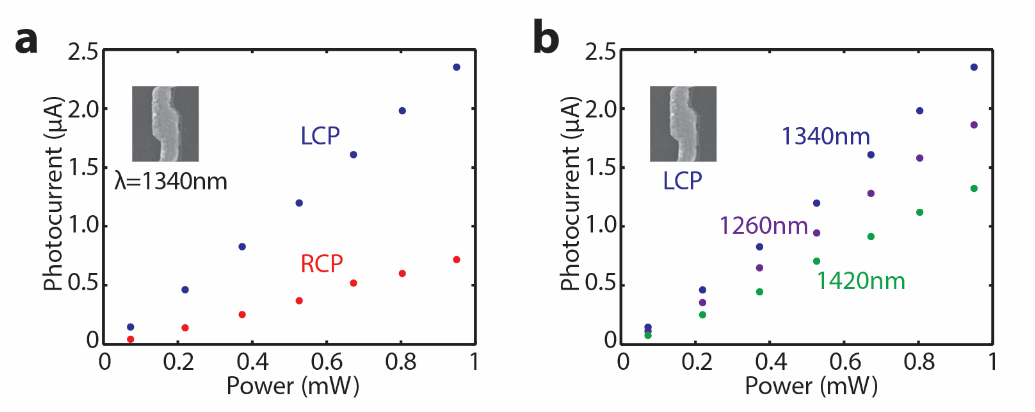

Supplementary Figure 11: Power dependent photocurrent for different polarization and wavelength. Unbiased photocurrent of the LH metamaterial was measured as a function of incident laser power for different polarization (**a**) and wavelength (**b**). Good linearity was observed in both cases, excluding any non-linear interactions. It also indicates the detector's ability to work over a wide range of incident power, overcoming saturation issues found in chiral organic semiconductors. The device area for this measurement is 70 μm x 70 μm.

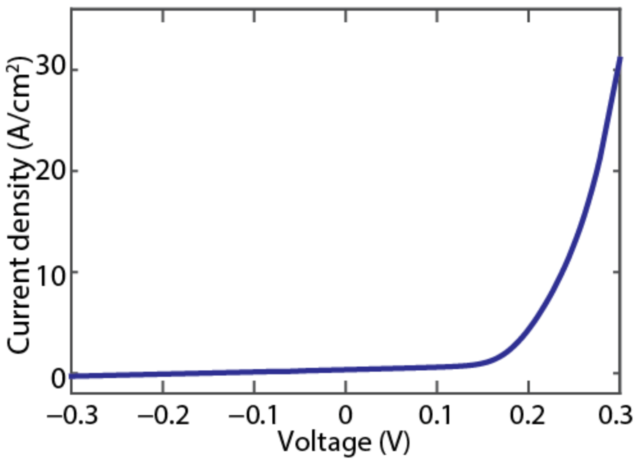

Supplementary Figure 12: Current-voltage characteristic curve of the device. Fitting this curve to thermionic emission theory[1], we can extract the Schottky barrier height to be 0.54 eV and the ideality factor to be 1.52.

Supplementary Notes:

Supplementary Note 1.

Understanding and designing strong circular dichroism in chiral metamaterials.

Due to the rotation of the electric field vector in circularly polarized light, direct relating the spectral position and magnitude of circular dichroism with the structure's geometry is usually challenging. Therefore, we will first investigate the system's optical response under linearly polarized light and then relate it to circularly polarized light, simply due to the fact that the CPL electric field vector can be decomposed into two perpendicular LPL electric field vectors, E_x and E_y, that are oscillating with a 90° phase shift, as shown in equation (1) and (2) of the main text.

First, we choose a system with an array of silver stripes without metal backplane aligned in the *x* direction with a unit cell shown in Supplementary Fig. 1a. To represent a more general situation, we choose the background material to be PMMA (n = 1.47) to avoid the substrate induced reflection and symmetry breaking. We examined the reflection coefficients of this system under two orthogonal linearly polarized light fields with electric field vectors of E_x and E_y. Both the amplitude and the phase of the reflected light from this system are shown in Supplementary Fig. 1c,d.

As can be seen in Supplementary Fig. 1c,d, since this system is anisotropic in the *xy* plane, both the amplitude and the phase of the two reflection coefficients $\boldsymbol{r}_{xx}$ and $\boldsymbol{r}_{yy}$ are different. Under CPL illumination (Supplementary Fig. 1e,f), the reflected field in the *x* direction, $\boldsymbol{r}_{xx}E_x$, for LCP and RCP are exactly the same whereas the reflected fields in the *y* direction, $\boldsymbol{r}_{yy}E_y$, have a 180° phase shift when comparing LCP to RCP. As a result, the total reflected field intensities

are the same for LCP and RCP and the material exhibits no circular dichroism (Supplementary Fig. 1b). This can also be confirmed by the fact that this system is non-chiral due to the existence of two mirror symmetry planes in the system.

To break the mirror symmetry of system, we cut two rectangles from the two diagonal corners of the silver stripe in the unit cell, forming a 'Z' shaped antenna as shown in Supplementary Fig. 2a. As a result, there is no mirror symmetry plane in the *xy* plane. Under linearly polarized light illumination (Supplementary Fig. 2c,d), one can see that this system is capable of rotating a linear polarization state into its orthogonal one. The polarization conversion coefficients are labeled as $\boldsymbol{r}_{xy}$ and $\boldsymbol{r}_{yx}$ and represent the conversion from E_y to E_x, and E_x to E_y, respectively. The polarization conversion coefficients for two orthogonal fields are identical ($\boldsymbol{r}_{xy} = \boldsymbol{r}_{yx}$). As a result, four reflected field components were observed: the unconverted scattered fields, $\boldsymbol{r}_{xx}E_x$, $\boldsymbol{r}_{yy}E_y$ and converted scattered fields, $\boldsymbol{r}_{yx}E_x$, $\boldsymbol{r}_{xy}E_y$ (Supplementary Fig. 2c,d).

When the structure is illuminated with circularly polarized light, the unconverted scattered field, $\boldsymbol{r}_{xx}E_x$, (or $\boldsymbol{r}_{yy}E_y$) will interfere with the converted scattered field, $\boldsymbol{r}_{xy}E_y$ (or $\boldsymbol{r}_{yx}E_x$). The reflected field with E_x or E_y polarization can be represented as,

$$E_{x,sum} = \boldsymbol{r}_{xx}E_x + \boldsymbol{r}_{xy}E_y \tag{1}$$

$$E_{y,sum} = \boldsymbol{r}_{yy}E_y + \boldsymbol{r}_{yx}E_x \tag{2}$$

Here, we use the vector field plots to illustrate the interference for both LCP and RCP. As can be seen in Supplementary Fig. 2e, for the case of LCP illumination (the phase of E_x is 90° ahead of E_y) the unconverted scattered field, $\boldsymbol{r}_{xx}E_x$, will destructively interfere with the converted scattered field, $\boldsymbol{r}_{xy}E_y$, resulting in a reduced total reflected field ($E_{x,sum}$). However, for the case of RCP illumination (the phase of E_y is 90° ahead of E_x) as shown in Supplementary

Fig. 2f, the unconverted scattered field, $\boldsymbol{r}_{xx}E_x$, will constructively interfere with the converted scattered field, $\boldsymbol{r}_{xy}E_y$, resulting in an enhanced total reflected field ($E_{x,sum}$). The asymmetric interference results in different absorption for LCP and RCP as shown in Supplementary Fig. 2b. As a result, a circular dichroism of about 0.35 for this single layer system is obtained.

To further enhance the circular dichroism, we added a metal ground plane and a dielectric spacer to the 'Z' shaped chiral metamaterial. Meanwhile, the structure's dimensions were adjusted to account for the different background materials (silicon and PMMA), the wavelength of interest (1200 nm to 1500 nm) and the fabrication resolution (blend angle) as shown in Supplementary Fig. 3a and Fig. 1a of the main text. The metal backplane blocks the transmission and forms a Fabry-Perot cavity leading to multiple reflections within the film. We tracked the reflected electric field evolution as a function of the number of reflections between the structure and the metal backplane as shown in Fig. 2e,f in the main text. The multiple reflections further enhance (LCP) or reduce (RCP) absorption, leading a circular dichroism up to 0.9 (Supplementary Fig. 3b). This can also be confirmed by investigating the reflection coefficients (Supplementary Fig. 3c,d) and the interference effects (Supplementary Fig. 3e,f) as we consider the single layer 'Z' shaped chiral metamaterial along with the metal ground plane as a single system. As can be seen in Supplementary Fig. 3e, for the case of LCP (E_x is 90° phase ahead of E_y), the unconverted scattered field $\boldsymbol{r}_{xx}E_x$, (or $\boldsymbol{r}_{yy}E_y$) will destructively interference with converted scattered field $\boldsymbol{r}_{xy}E_y$ (or $\boldsymbol{r}_{yx}E_x$), resulting in a reduced total reflected field for both $E_{x,sum}$ and $E_{y,sum}$. An opposite situation is observed for the case of RCP (E_x is 90° phase after E_y) as shown in Supplementary Fig. 3f. The strong asymmetric interference effect results in

different absorption for LCP and RCP. As a result, a circular dichroism of about 0.9 is obtained as shown in Supplementary Fig. 3b.

In an ideal case, under LCP illumination, the unconverted scattered field, $\boldsymbol{r}_{xx}E_x$, (or $\boldsymbol{r}_{yy}E_y$) should be exactly out of phase (180° phase shift) with the converted scattered field $\boldsymbol{r}_{xy}E_y$ (or $\boldsymbol{r}_{yx}E_x$) while maintaining the same amplitude to completely cancel out the reflected field. Whereas for the case of RCP, the unconverted scattered field, $\boldsymbol{r}_{xx}E_x$, (or $\boldsymbol{r}_{yy}E_y$) should be exactly in phase (0° phase shift) with the converted scattered field, $\boldsymbol{r}_{xy}E_y$ (or $\boldsymbol{r}_{yx}E_x$), to achieve maximum reflection. The field vectors under these ideal conditions are illustrated in Supplementary Fig. 4.

From Supplementary Fig. 4, we can summarize the ideal conditions for maximizing circular dichroism (maximizing LCP absorption, minimizing RCP absorption) in this planar chiral metamaterial as:

$$\left|\boldsymbol{r}_{xx}\right| = \left|\boldsymbol{r}_{xy}\right| = \left|\boldsymbol{r}_{yx}\right| = \left|\boldsymbol{r}_{yy}\right| \tag{3}$$

$$\phi_{xx} - 90^0 = \phi_{xy} = \phi_{yx} = \phi_{yy} - 270^0 \tag{4}$$

For the case of maximizing RCP absorption and minimizing LCP absorption, the ideal conditions will be:

$$\left|\boldsymbol{r}_{xx}\right| = \left|\boldsymbol{r}_{xy}\right| = \left|\boldsymbol{r}_{yx}\right| = \left|\boldsymbol{r}_{yy}\right| \tag{5}$$

$$\phi_{xx} + 90^0 = \phi_{xy} = \phi_{yx} = \phi_{yy} + 270^0 \tag{6}$$

These conditions set several requirements for the planar metamaterial. First, the metamaterial needs to possess linear polarization conversion to produce the converted field. Second, the metamaterial needs to be anisotropic in order to produce the phase difference. Third, the metamaterial needs to be lossy. Any planar metamaterial that fulfills these three requirements will exhibit circular dichroism.

It should be noted that the ‘Z’ shaped antenna array was chosen due to the fact that the array needs to be electrically connected for hot electron photodetection. There are many alternative geometries that can produce the same optical properties. For example a 45° tilted dipole antenna[2] can be combined with vertically aligned dipole antennas or three dipole antennas with varying relative displacements could be used. Each of these geometries can result in a circular dichroism up to 0.9. The circular dichroism is only limited by the material damping of the plasmonic materials.

Supplementary Note 2. Circular conversion dichroism.

Here, we reexamine the proposed chiral metamaterial that is shown in Fig.1a of the main text and Supplementary Fig. 3a. At the resonant frequency under RCP illumination (E_y is 90° phase ahead of E_x) the phase of the reflected field, $E_{x,sum}$, will be about 90° ahead of $E_{y,sum}$ while the amplitudes of the two beams are roughly identical (Supplementary Fig. 3f). As a result, the total reflected field becomes LCP. Therefore, there is a strong circular polarization conversion from RCP to LCP. However, in the case of LCP illumination, due to the destructive interference, conversion from LCP to RCP is not observed. Therefore, the strong asymmetric interference can also be viewed as the strong asymmetric conversion between LCP and RCP[3,4], or circular conversion dichroism, as shown in Supplementary Fig. 5.

Supplementary Note 3. Chiral-selective absorption in semiconductor

It should be noted that the ability of distinguishing and detecting circularly polarized light can also be achieved by integrating a chiral metamaterial and a regular photodetector, leading to chiral-selective absorption and photocurrent in the semiconductor. However, this approach does

not necessarily produce high polarization selectivity even though higher quantum efficiency can be realized. To further expand on this point, we integrated our chiral metamaterial design with a semiconductor photodetector and investigated the direct absorption in the semiconductor. Specifically, we replaced the PMMA dielectric spacer with germanium, which serves as the semiconductor. We examined the total absorption as well as the absorption in germanium for LCP and RCP light, the simulation results are shown in Supplementary Fig. 7.

As we can see from Supplementary Fig. 7, the chiral metamaterial integrated with germanium can be designed to have the same type of resonance as we show in Fig.2 of the main text. However, the absorption difference in germanium for LCP and RCP light is less than 0.05 at the resonance wavelength and the polarization selectivity is only about 2.5 based on the simulation. As a comparison, the simulated polarization selectivity of the chiral metamaterial (Fig.2 in the main text) is about 10. The relatively low selectivity, compared to hot electron injection, is mainly due to two reasons: 1) for the resonant polarization state, there is competition between the semiconductor absorption and the metal absorption. 2) for the non-resonant polarization state there is still non-negligible direct absorption in the semiconductor.

Supplementary Note 4. On the structure of the photo injection current.

As can be seen in Supplementary Fig. 10, we plot the normalized absorption and photo-current spectra to compare their shapes. We see an interesting and strong effect of the asymmetry of the spectra. Both peaks in the absorption and photo-current are somewhat asymmetric. The absorption peak exhibits relatively weak asymmetry and is slightly larger on the red side, whereas the photo-current peak is clearly asymmetric and clearly smaller on the red side. Using our modeling, we can understand such asymmetries. The almost symmetric absorption peak

comes from the strong plasmon resonance and was calculated from the Maxwell's equations incorporating the smooth Drude-like dielectric function of metal in this wavelength interval. The asymmetry of the photo-current peak comes mostly from the involvement of the Fowler law in the equation for the photo-current given in the Methods section. This equation for the photo-current has the field factor and the Fowler-law factor, $(\hbar\omega - \Delta\phi_{Barrier})^2$, that is a strongly asymmetric function of the wavelength. The Fowler law takes into account the fact that some of the generated hot electrons do not have the proper momentum and energy to cross the interface into the silicon.

Supplementary References

1. Sze, S. M. & Ng, K. K. in *Physics of Semiconductor Devices, 3rd Edition.; John Wiley & Sons, Inc.; NJ* 164, 682 (2007).

2. Grady, N. K. *et al.* Terahertz Metamaterials for Linear Polarization Conversion and Anomalous Refraction. *Science* **340,** 1304–1307 (2013).

3. Fedotov, V. *et al.* Asymmetric Propagation of Electromagnetic Waves through a Planar Chiral Structure. *Phys. Rev. Lett.* **97,** 167401 (2006).

4. Fedotov, V. A., Schwanecke, A. S., Zheludev, N. I., Khardikov, V. V. & Prosvirnin, S. L. Asymmetric transmission of light and enantiomerically sensitive plasmon resonance in planar chiral nanostructures. *Nano Lett.* **7,** 1996–1999 (2007).
